# Successful Treatment with Bimekizumab of a Psoriatic Patient Undergoing Hemodialysis: A Case Report and Review of the Literature

**DOI:** 10.3390/jcm13082250

**Published:** 2024-04-12

**Authors:** Nicoletta Bernardini, Luca Ambrosio, Ersilia Tolino, Ilaria Proietti, Nevena Skroza, Concetta Potenza

**Affiliations:** 1 Department of Medical-Surgical Sciences and Biotechnologies, Dermatology Unit “Daniele Innocenzi”, “Sapienza” University of Rome, Polo Pontino, 04100 Latina, Italy; nicoletta.bernardini@libero.it (N.B.); ersiliatolino@gmail.com (E.T.); proiettilaria@gmail.com (I.P.); nevena.skroza@uniroma1.it (N.S.); concetta.potenza@uniroma1.it (C.P.); 2 Dermatology Unit, Department of Clinical Internal Anesthesiologic Cardiovascular Sciences, “Sapienza” University of Rome, 00185 Rome, Italy

**Keywords:** psoriasis, bimekizumab, hemodialysis

## Abstract

**Background/Objectives**: Treating psoriasis patients requires the consideration of potential underlying complications like latent viral infections and chronic kidney disease, which may influence therapy selection. **Case presentation**: A patient with end-stage kidney disease (ESKD) undergoing hemodialysis (HD) was successfully treated with bimekizumab, an IgG1 humanized monoclonal antibody inhibiting interleukin (IL)-17A and IL-17F. This case appears to be the first documented instance of effective anti-IL-17A/IL-17F antibody treatment in a psoriasis patient undergoing HD, with a sustained positive response for eight months. **Discussion**: Studies indicate the comparable pharmacokinetics, efficacy, and safety of certain psoriasis drugs in patients with chronic kidney disease (CKD) and those with normal renal function. The positive clinical outcome observed following treatment with bimekizumab aligns with the existing literature on this topic. However, further studies are needed to objectively evaluate the pharmacokinetics, efficacy, and safety of this drug in this specific setting. **Conclusions**: This documented case represents the first known use of bimekizumab to treat psoriasis in patients undergoing dialysis, suggesting its potential effectiveness and safety in this population.

## 1. Introduction

Psoriasis is a chronic, immune-mediated, inflammatory skin disease with a chronic-relapsing course that significantly impacts the quality of life of affected patients. It is a systemic disease, with approximately one-third of patients also presenting or developing concomitant psoriatic arthritis [1]. The study of the pathogenetic mechanisms underlying this condition has identified several of the pathways and cytokines involved, such as IL-17 and IL-23 [2]. Consequently, these cytokines, plus tumor necrosis factor (TNF) and interleukin (IL)-12/23, have become the target of approved biological therapies for this condition [3]. The IL-17 axis, mediated by IL-17A and IL-17F, plays a crucial role in the pathogenesis of psoriasis. These cytokines, which can form homodimers and heterodimers, act on the common receptor complex IL-17RA/RC and are both overexpressed in psoriatic lesions [4]. Consequently, several therapies targeting IL-17A or the IL-17RA receptor subunit have been developed and prove effective in managing patients with this condition [5,6,7]. However, literature data suggest differences in the regulation of these molecules, with IL-17F being more prominently expressed in lesional skin compared to IL-17A [8]. Bimekizumab represents the first monoclonal antibody to comprehensively inhibit the IL-17A and IL-17F pathways by targeting the IL-17A/A, IL-17A/F, and IL-17F/F isoforms. The simultaneous inhibition of both isoforms has been linked to a more significant reduction in inflammatory cytokines and neutrophil chemotaxis compared to the inhibition of IL-17A alone [9]. Currently, bimekizumab is approved for the treatment of psoriasis [10], psoriatic arthritis [11,12], and axial spondyloarthritis [13]. Clinical trials are underway to evaluate its efficacy and safety in hidradenitis suppurativa, with encouraging results [14]. In the treatment of psoriasis patients, it is crucial to consider potential underlying complications/comorbidities, such as latent viral infections and chronic kidney disease, as they could impact the selection of appropriate therapy [15].

## 2. Case Report

**Figure 1:**
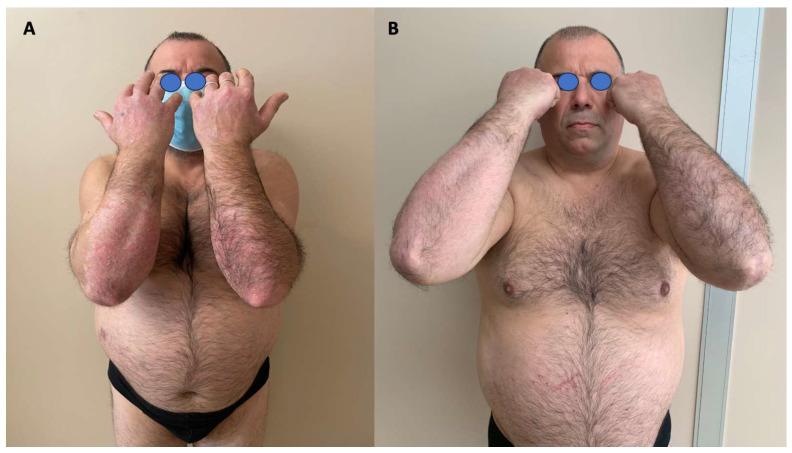
(**A**) Thick erythematous scaly plaques on the upper limbs before bimekizumab treatment T0; (**B**) complete resolution of the lesions 4 weeks after treatment with bimekizumab.

A male patient aged 43 sought treatment at our chronic inflammatory disease outpatient clinic with a complaint of a widespread rash that had not responded to topical treatment for approximately four months. The patient had a medical history of psoriasis since the age of 10, a family history of psoriasis (father), and had been diagnosed with end-stage kidney disease due to hypertensive nephropathy about four years prior. The patient was currently undergoing hemodialysis treatment three times per week at a separate facility and was awaiting organ transplantation. Physical examination revealed erythematous scaly plaques on the dorsal surfaces of the upper and lower limbs, accompanied by smaller lesions located on the trunk and back (Figure 1). The patient reported itching, with a Psoriasis Area Severity Index (PASI) of 11, a Body Surface Area (BSA) of 30, a Numerical Rating Scale (NRS) pruritus score of 6, a NRS pain score of 0, and a Dermatology Life Quality Index (DLQI) of 18. Joint pain was not present. Additionally, nodular and plaque-like lesions were identified at the injection site on the patient’s right forearm, where hemodialysis was performed. The patient’s diagnosis of psoriasis was confirmed through a skin biopsy, which revealed characteristic histopathological features such as hyperkeratosis with parakeratosis, acanthosis, papillomatosis, dilated vessels at the apex of the dermal papillae, and inflammatory infiltrate. Following the exclusion of ongoing infections and organ abnormalities via hematochemical screening, and in consultation with the patient’s nephrologist, it was decided to initiate treatment with bimekizumab, a monoclonal antibody targeting IL-17A and IL-17F. This decision was based on the existing literature on other drugs in the same class, which showed positive outcomes and efficacy in treating psoriasis patients with similar comorbidities [16,17,18,19,20]. The treatment plan for bimekizumab involves administering two pre-filled 160 mg pens (320 mg) every four weeks for a total of 16 weeks, followed by a maintenance regimen of 320 mg every eight weeks. Following the initial administration of the drug, the patient reported a significant improvement in pruritus symptoms (pNRS: 2) and a minor improvement in skin lesions (PASI: 4.4) after one week. One month later, at the time of the second administration, the patient achieved complete clearance of skin lesions and a notable improvement in his overall quality of life (PASI: 0; BSA: 0; NRS pruritus: 3; DLQI: 4); (Figure 1). The patient’s positive response to bimekizumab therapy was sustained for eight months following the start of treatment (follow-up duration at the time of publication), with no significant changes observed in renal function as assessed by regular evaluations performed by the patient’s HD center.

## 3. Discussion

Dialysis can have a significant impact on the natural history of psoriasis, affecting both its clinical course and the risk of onset or exacerbation. This impact is thought to be mediated by the release of various pro-inflammatory cytokines, particularly Th1-type cytokines such as IL-1, IL-6, IL-8, IL-12, and TNF-α, which are associated with immune dysregulation and inflammation [20,21]. A retrospective cohort study involving 74,916 patients with ESRD undergoing chronic HD demonstrated a significantly higher incidence rate of psoriasis in this population compared to healthy controls. Furthermore, HD patients tended to develop psoriasis more rapidly than individuals in the control group. However, the relative risk of developing psoriatic arthritis was not found to be increased in patients undergoing hemodialysis compared to the controls [22]. Additionally, research has shown that patients with ESRD and psoriasis tend to be hospitalized at a younger age, on average, compared to those with ESRD alone. This suggests that the presence of psoriasis may exacerbate the clinical course of ESRD and increase the need for medical intervention or hospitalization in affected individuals [23]. Moreover, the potential for the clinical remission of psoriasis following kidney transplantation is an important consideration in the management of patients with ESRD undergoing HD who also have psoriasis. The literature indeed reports cases of psoriasis improvement or even complete remission post-kidney transplantation [24]. This remission is believed to be primarily due to the immunosuppressive therapy administered to prevent organ rejection, particularly oral calcineurin inhibitors such as tacrolimus and cyclosporine. These medications work by suppressing the immune system, thereby reducing inflammation and the autoimmune response characteristic of psoriasis [24]. However, it is important to carefully weigh the risks and benefits of transplantation, considering factors such as the patient’s overall health status, comorbidities, and eligibility for transplantation, in collaboration with a multidisciplinary medical team. Another retrospective cohort study involving 8911 patients emphasized the heightened risk of infections in individuals with both psoriasis and ESRD, attributed not only to the underlying renal dysfunction but also to the altered function of the epidermal barrier and the presence of permanent venous accesses associated with hemodialysis [25]. Furthermore, this study highlighted the potential benefits of appropriate therapeutic management of psoriasis in this population. The effective control of psoriasis has been associated with a reduction in the risk of infections and an improvement in overall survival among patients with ESRD. This underscores the importance of the proactive management of psoriasis in patients undergoing hemodialysis to mitigate the risk of infections and enhance long-term outcomes [25]. Currently, the majority of available data on therapy with biologic drugs in this specific setting primarily stem from case reports and case series focusing on the treatment of individuals with psoriasis undergoing hemodialysis (HD). This reliance on case reports and case series is largely due to the rarity of this condition and the limited availability of randomized controlled clinical trials specifically examining the efficacy and safety of biologic drugs in this population. However, despite their limitations, case reports and case series can offer valuable clinical insights into the use of biologic drugs in patients with psoriasis undergoing HD. These studies can help us to better understand treatment responses, adverse effects, and any potential complications that may arise in this particular population. The analysis of the literature on the treatment with biologic drugs in patients with psoriasis and end-stage renal disease (ESRD) undergoing hemodialysis reveals valuable insights from 23 cases (Table 1). Among these cases, 17 provide comprehensive data on various clinical aspects, treatment response, and adverse events. In this subset of 17 cases, the majority of patients are male (14 out of 17), with a mean age of 56 years. Psoriasis predominantly affects areas such as the scalp, trunk, and limbs, with 5 cases presenting with erythroderma. Before initiating biologic therapy, the mean PASI score stands at 25.7. However, following treatment with biologics, the mean PASI significantly decreases to 1.3, underscoring the efficacy of these therapies in this specific population. The mean follow-up duration is 16.4 months, allowing for adequate assessment of treatment outcomes over time. Among the patients, a notable proportion (4 out of 17) presents with concomitant psoriatic arthritis, all of whom respond favorably to biologic therapy. Importantly, the majority of patients (15 out of 17) are biologic-naive, highlighting the effectiveness of these agents as first-line treatment options. Only one case reports adverse effects following treatment with brodalumab, manifesting as a chronic cough. Overall, these findings underscore the potential of biologic therapy in effectively managing psoriasis in patients with ESRD undergoing hemodialysis, with notable improvements in disease severity and limited adverse events observed in this population. Bustos et al. reported the case of a 65-year-old man with hypertension, ischemic heart disease, and chronic renal failure diagnosed four years ago. He had previously been managed with methotrexate, cyclosporine, acitretin, phototherapy, and etanercept with little benefit. Under treatment with the anti-IL 12/23 ustekinumab, the patient achieved PASI 100 from an initial value of 15 and maintained his response to therapy at a distance of 17 months of follow-up without side effects [26]. Cassano et al. described the case of a 69-year-old man with psoriasis and psoriatic arthritis successfully treated with anti-TNF-alfa etanercept, reducing his PASI score from 21 to 8 after 6 months, with a clear improvement in the joint component as well [27]. Ikuma et al. published the case of a 60-year-old woman with erythrodermic psoriasis treated with anti-IL-17A secukinumab, showing partial improvement in the PASI score from 49.8 to 14.8 at 28 months of follow-up without any side effects [28]. Ishiabashi et al. reported the case of a 60-year-old subject with a PASI score of 39.6 undergoing hemodialysis for 2 months, treated with the anti-IL-17R brodalumab, achieving and maintaining PASI 100 at 12 months of follow-up. Four months after the start of therapy, the patient complained of a chronic cough attributed to fluid overload, which was resolved with appropriate dietary and weight control [19]. Koike et al. reported the case of a 48-year-old man with psoriasis and psoriatic arthritis which was successfully managed with anti-IL-17A ixekizumab, achieving PASI 0 from a baseline PASI of 21.6 and maintaining at 18 months of follow-up without experiencing any side effects [18]. Kusakari et al. described a case of severe psoriasis successfully managed with adalimumab therapy. The patient had previously been treated with cyclosporine, infliximab, and ustekinumab. Infliximab therapy resulted in pulmonary edema due to hydration during the intravenous injection, while ustekinumab therapy did not lead to clinical improvement. Following adalimumab therapy, the patient achieved and maintained PASI 100 from a baseline value of 42.3 without experiencing any side effects [29]. Larquey et al. reported a case of psoriasis affecting the scalp, trunk, limbs, auditory canal, and gluteal area in a patient on hemodialysis for 7 years, who had previously been treated only with topical agents. The patient experienced clinical improvement following ustekinumab therapy, with a decrease in PASI from 13.6 to 4.5 [30]. Mukai et al. published a case series regarding three patients with psoriasis and ESRD on dialysis who were successfully treated with secukinumab. Two of these patients had erythrodermic psoriasis, and one of them was undergoing peritoneal dialysis. The third patient also had psoriatic arthritis. In all three cases, PASI 90 was achieved and maintained without any side effects [17]. Nimmanitya et al. described the case of a patient with psoriasis without joint involvement, successfully managed with ustekinumab therapy, maintaining the outcome at 33 months of follow-up [31]. Pizzatti et al. reported the case of an erythrodermic patient who achieved and maintained PASI 100 at a remarkable 52 months from the start of secukinumab therapy. This represents the case with the longest available follow-up in the literature and confirms the efficacy of such treatments not only in the short but also in the long term, crucial for a chronic relapsing disease like psoriasis. Pizzatti et al. reported the case of an erythrodermic patient who achieved and maintained PASI 100 at 52 weeks from the start of secukinumab therapy [32]. Saougu et al. published the case of a 52-year-old patient with psoriasis and psoriatic arthritis successfully treated for both components with intravenous infliximab infusions [33]. Shibata et al. reported the case of a patient with psoriasis undergoing hemodialysis who achieved and maintained PASI 100 at 24 months from the start of secukinumab therapy [34]. Finally, Umezawa et al. published a case series involving three patients with psoriasis and end-stage renal disease undergoing hemodialysis, successfully treated with ustekinumab, with maintenance of the response at 12 months [35]. These data provide compelling evidence for the efficacy and safety of biological treatments in the management of psoriasis in patients with ESRD undergoing hemodialysis. Among the 16 patients analyzed, the majority achieved significant improvements in their Psoriasis Area and Severity Index (PASI) scores: 9 patients attained and maintained PASI 100, indicating complete clearance of psoriasis symptoms, while 6 patients reached PASI 90, reflecting a high level of improvement. Only one patient, treated with secukinumab, achieved PASI 70, indicating a substantial reduction in disease severity. Importantly, the diverse classes of biologic drugs utilized—including anti-TNF-alpha agents, anti-IL-12/23 therapy, IL-17A inhibitors, and IL-17R blockers—all demonstrated efficacy in this patient population. This underscores the versatility of these treatments and the importance of tailoring therapy to individual patient needs. Furthermore, the identification of cyclosporine as a potential triggering cause of ESRD in five patients underscores the importance of the vigilant monitoring of renal function and blood pressure in psoriasis patients receiving this medication. These findings emphasize the need for the careful consideration of the risks and benefits associated with prolonged cyclosporine therapy and highlight the importance of exploring alternative treatment options to minimize renal-related complications in susceptible individuals.

The positive outcome observed in our case treated with bimekizumab aligns with the existing literature on the efficacy of biological drugs in treating patients with psoriasis undergoing HD and other comorbidities. However, a limitation is represented by the lack of data regarding safety and pharmacokinetics such as IL-17A/F levels before and after treatment and hemodialysis therapy, which could have further validated the clinical outcomes obtained. Studies have shown that the pharmacokinetics, efficacy, and safety of certain drugs used in the treatment of psoriasis are comparable in patients with chronic kidney disease (CKD) and those with normal renal function. Furthermore, hemodialysis does not appear to significantly impact drug clearance in these patients [16]. There are two main reasons why antibody-based drugs, such as bimekizumab, are not expected to be cleared by hemodialysis or affected by renal impairment. Firstly, these drugs are typically broken down through intracellular catabolism, which means that their biological half-life is around 14 to 21 days, and they are not cleared through the kidney or liver. Secondly, biological agents are large, high-molecular-weight proteins that are unlikely to be cleared by HD due to their size [18]. According to a study by Larquey et al., only one patient who received ustekinumab showed a decrease in plasma concentration of the therapeutic antibody. This could be related to the fact that ustekinumab has a longer half-life compared to other biological drugs used to treat psoriasis, which means that the number of dialysis sessions between two injections is more significant, and the impact on the elimination of the molecule may be increased [20].

## 4. Conclusions

As far as we know, this is the first documented case of using bimekizumab to treat psoriasis in patients undergoing dialysis. The results suggest that bimekizumab could be an effective treatment for severe psoriasis in this patient population. Overall, these findings highlight the complex interplay between renal function, inflammation, and immune dysregulation in the context of psoriasis, particularly in patients undergoing hemodialysis for ESRD. Further research is needed to objectively evaluate the pharmacokinetics, efficacy, and safety of this drug in this specific setting and to optimize management strategies for individuals with both psoriasis and renal disease.

## Figures and Tables

**Table 1:** Clinical features, PASI scores, adverse events in patients affected by psoriasis and end stage renal disease undergoing hemodialysis treated with biologics.

Author	Year	Treatment	PASI T_0_	PASI T_1_	Follow-Up	Psoriatic Arthritis	Previous Therapies	Additional Info	Adverse Events
Bustos et al. [26]	2014	Ustekinumab	15	0	17 months	no	Methotrexate, cyclosporine, acitretin, phototherapy, Etanercept	hypertension, ischemic heart disease, and chronic renal failure four years ago	No
Cassano et al. [27]	2008	Etanercept	21	0.8	6 months	yes (effective)	Topical, phototherapy, cyclosporine	HD since 9 years, atrial fibrillation; asymptomatic HCV infection	No
Ikuma et al. [28]	2019	Secukinumab	49.8	14.8	28 months	no	Topical	HD since 39 y.o.	No
Ishiabashi et al. [19]	2019	Brodalumab	39.6	0	12 months	no	Cyclosporine	HD since 2 months; diabetes	Chronic cough
Koike et al. [18]	2019	Ixekizumab	21.6	0	18 months	yes	none	HD since 4 year, HBV carrier; improvement of the psoriatic arthritis	No
Kusakari et al. [29]	2015	Adalimumab	42.3	0	12 months	no	Cyclosporine, infliximab (AE), ustekinumab (no improvement)	Pulmonary edema because of hydration during the i.v. injection of infliximab	No
Larquey et al. [20]	2017	Adalimumab	-	-	-	no	-	-	No
Larquey et al. [20]	2017	Etanercept	-	-	-	no	-	-	No
Larquey et al. [20]	2017	Etanercept	-	-	-	no	-	-	No
Larquey et al. [20]	2017	Etanercept	-	-	-	no	-	-	No
Larquey et al. [30]	2014	Ustekinumab	13.6	4.5	9 months	no	Topical	HD since 7 years, obesity	No
Larquey et al. [20]	2017	Ustekinumab	-	-	-	no	-	-	No
Mukai et al. [17]	2019	Secukinumab	33	3.3	5 months	yes	Phototherapy	Peritoneal dyalisis since 12 years	No
Mukai et al. [17]	2019	Secukinumab	31	3.1	4 months	no	Phototherapy	-	No
Mukai et al. [17]	2019	Secukinumab	-	-	-	yes (effective)	Cyclosporine	-	No
Nimmanitya et al. [31]	2016	Ustekinumab	15.4	2.8	33 months	no	Topical, phototherapy	Policystic kidney disease	No
Pizzatti et al. [32]	2020	Secukinumab	31.5	0	12 months	no	Cyclosporine, phototherapy	-	No
Saougu et al. [33]	2010	Infliximab	35.1	0	6 months	yes (effective)	none	improvement of the psoriatic arthritis	No
Shibata et al. [34]	2018	Secukinumab	22	0	24 months	no	Topical, oral corticosteroids	-	No
Umezawa et al. [35]	2015	Ustekinumab	10.4	0	12 months	no	Topical	HD since 10 years	No
Umezawa et al. [35]	2015	Ustekinumab	12.4	3	12 months	no	Topical	HD since 10 years	No
Umezawa et al. [35]	2015	Ustekinumab	17.2	2	12 months	no	Topical, phototherapy	HD since 1 year	No

## Data Availability

Dataset available upon request from the authors.
